# Splenic rupture secondary to diverticulitis: a case report

**DOI:** 10.1093/jscr/rjaf442

**Published:** 2025-06-25

**Authors:** Muhi Dean Barazi, Malek Zanbrakji, Patrick Stone, Essam Tellawi, Fares Chamma, Ahmad Safra

**Affiliations:** Department of Biosciences, Rice University, 6100 Main Street, Houston, TX 77005, United States; Department of Biosciences, Georgetown University, 37th and O Streets, Northwest Washington, DC 20057, United States; Department of Vascular Surgery, Vanderbilt University School of Medicine, 1161 21st Avenue South, Nashville, TN 37232, United States; Department of Gastroenterology, University of Michigan Health-Sparrow, 1200 East Michigan Avenue, Suite 715, Lansing, MI 48912, United States; Department of Cardiovascular Research, Indiana University School of Medicine, 1801 N Senate Avenue, Indianapolis, IN 46202, United States; Department of Transplant Surgery, INOVA Fairfax Hospital, 3300 Gallows Road, Falls Church, VA 22042, United States

**Keywords:** atraumatic splenic rupture, spontaneous splenic rupture, diverticulitis

## Abstract

Atraumatic splenic rupture (ASR) is a rare condition that may be idiopathic or may occur in the setting of certain inflammatory processes, infections, malignancies, and autoimmune conditions. Here, we report a very rare case of ASR secondary to complicated diverticulitis. A 49-year-old female presented with sudden onset of severe abdominal pain on the day after discharge, following attempted non-operative treatment of perforated diverticulitis at the splenic flexure. She denied any history of trauma. Computed tomography showed new splenic subcapsular hematoma and gas consistent with splenic rupture. Exploratory laparotomy with splenectomy, distal pancreatectomy, splenic flexure resection, and end transverse colostomy was performed, and the patient recovered well. Although it is rare, ASR can be life threatening, and clinicians should be aware of its association with diverticulitis.

## Introduction

Rupture of the spleen in the absence of trauma, called spontaneous or atraumatic splenic rupture (ASR), is rare, occurring in ~0.5% to 3% of all cases of rupture of the spleen [1, 2]. ASR can occur in the setting of localized inflammatory processes, infections, hematologic malignancies, and autoimmune conditions, among others, or it may be idiopathic [3, 4]. Diverticulitis is a common indication for elective resection of the colon and leads to nearly 200 000 inpatient admissions each year in the United States [5]. Complications, including perforation, abscess, obstruction, and fistulization, occur in ~12% of patients with diverticulitis [5]. While the literature on ASR is limited, diverticulitis is an extremely rare cause [3, 4]. Herein, we report a case of ASR in the setting of complicated diverticulitis of the splenic flexure.

## Case report

A 49-year-old female presented to the emergency department complaining of left upper quadrant abdominal pain, nausea, fever, and chills for the past 24 h. The patient had a history of a prior episode of perforated sigmoid diverticulitis 1 year ago that was medically managed.

**Figure 1:**
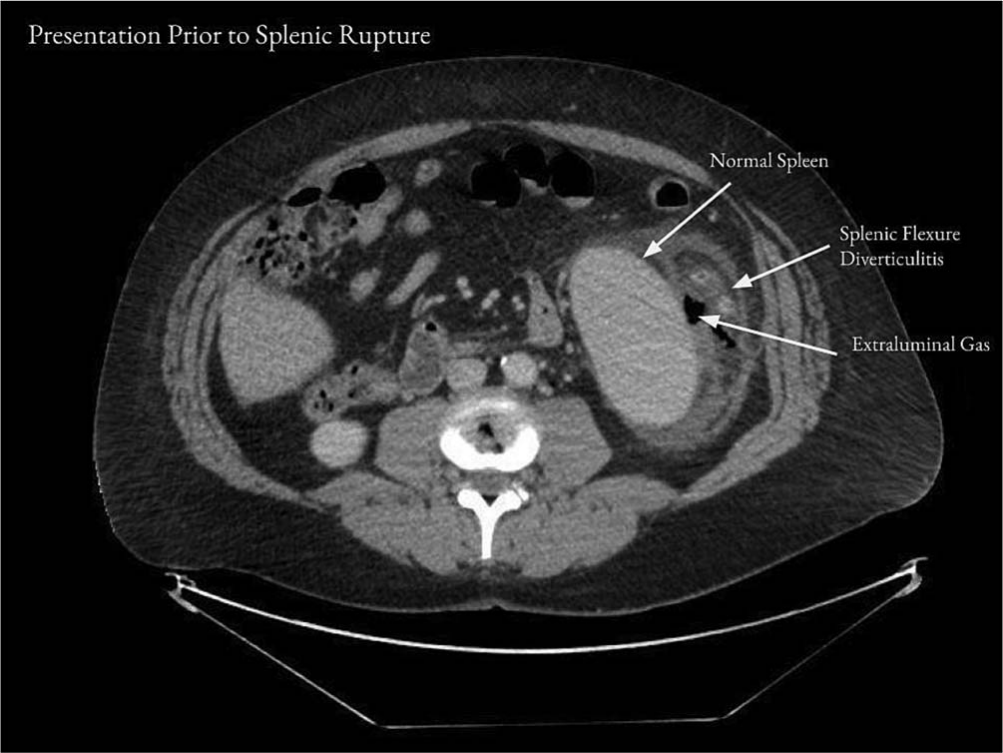
Computed tomography of the abdomen and pelvis on presentation showed acute diverticulitis of the splenic flexure with moderate wall thickening and adjacent extraluminal gas consistent with localized perforation.

On examination, the patient appeared mildly ill and uncomfortable. Blood pressure was 132/81, heart rate was 107, and temperature was 37.8°C. The abdomen was soft, with moderate left upper quadrant and left flank tenderness, mild distention, and no guarding. Laboratory workup revealed a white blood cell (WBC) count of 13.2 × 10^3^/μl, a hemoglobin of 13.5 g/dl, and a hematocrit 36.0%. Computed tomography (CT) of the abdomen and pelvis with contrast showed acute diverticulitis of the splenic flexure with moderate wall thickening. There was adjacent extraluminal gas consistent with localized perforation (Fig. 1).

The patient was admitted to the colorectal surgery service with broad spectrum intravenous antibiotics. Over the following several days, the patient’s abdominal pain and tenderness improved, and the WBC decreased to 9.1 × 10^3^/μl. Surgical intervention was not recommended given that her two episodes of diverticulitis occurred at different locations within the colon. The patient was discharged home on oral antibiotics on the third hospital day.

**Figure 2:**
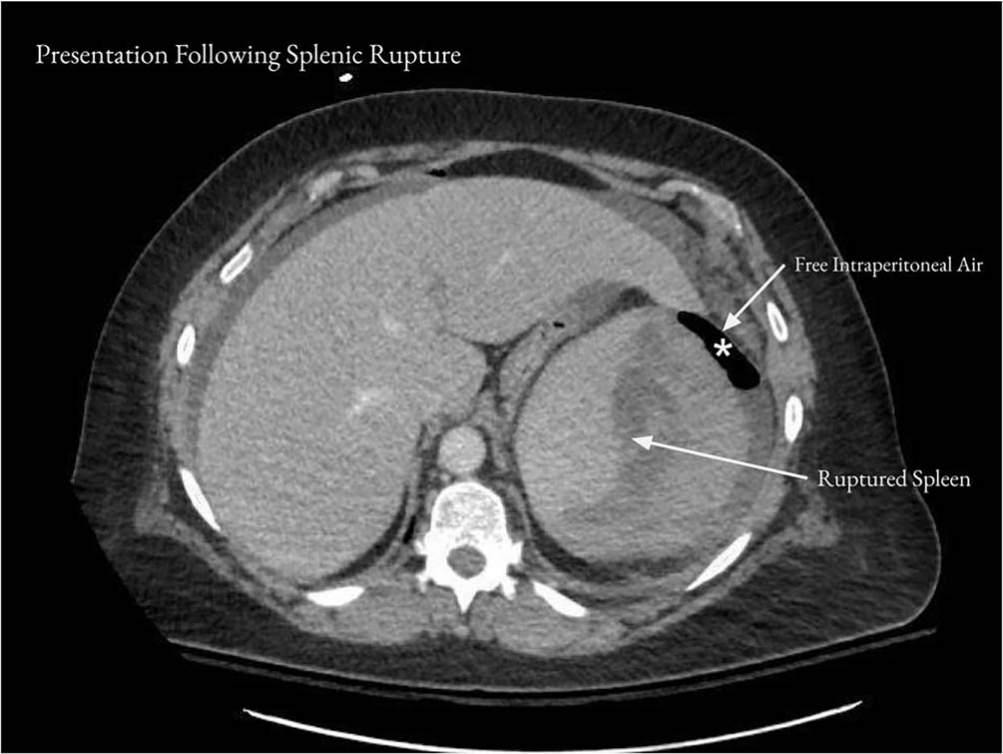
After the patient represented with acute worsening of abdominal pain, CT of the abdomen and pelvis showed a 7.2 cm subcapsular hematoma of the spleen (arrows) and moderate free intraperitoneal air (asterisk).

On the day following discharge, the patient presented to the emergency department complaining of sudden worsening of her abdominal pain. She reported that she had been sitting on her couch when she felt a “pop” followed by severe left upper quadrant pain, chills, and nausea. On examination, heart rate was in the 110 s, and there was mild abdominal distention and moderate tenderness to palpation over the left upper quadrant. WBC was 12.2 × 10^3^/μl, hemoglobin was 9.6 g/dl, and hematocrit was 26.7%. CT of the abdomen and pelvis showed a new subcapsular hematoma of the spleen, measuring 7.2 cm in thickness, with adjacent subcapsular gas (Fig. 2). There was moderate free intraperitoneal air.

The patient underwent urgent exploratory laparotomy and splenectomy. The spleen was noted to be enlarged with a large subcapsular hematoma, inflammatory changes, and an abscess cavity. The tail of the pancreas was adherent to the spleen, necessitating a distal pancreatectomy. The splenic flexure was then resected; a small hole that had perforated directly onto the splenic capsule was identified in its upper posterior aspect. There was adjacent pus, fibrinous exudate, and a small amount of solid stool. Due to diverticular disease of the sigmoid colon, an end transverse colostomy was performed. Histopathology of the spleen showed massive splenomegaly with focal capsular disruption and subcapsular and intraparenchymal hemorrhage. The splenic flexure demonstrated perforated diverticulitis with diffuse intramural and subserosal abscess formation.

The patient tolerated the procedure well and recovered well over the subsequent week. She was discharged on postoperative Day 7 with home intravenous antibiotics and planned follow-up in 2 weeks.

## Discussion

The spleen is one of the most commonly injured organs in blunt abdominal trauma [6]. Rupture of the spleen in the absence of trauma is very rare but has been reported in association with a variety of underlying conditions [3, 4]. In a systematic review of 845 patients with ASR, the most common causes included hematologic malignancies (e.g. acute leukemia, non-Hodgkin’s lymphoma), viral infections (e.g. mononucleosis, cytomegalovirus), and local inflammatory conditions (e.g. pancreatitis) [3]. In ~7% of cases, no underlying cause was identified [3]. In another systematic review of 613 cases of ASR, the most commonly identified causes were infectious (e.g. malaria, mononucleosis), hematologic (neoplastic and non-neoplastic), and non-hematologic neoplastic (e.g. angiosarcoma, choriocarcinoma) [4]. Other predisposing factors included rheumatologic disorders, amyloidosis, pregnancy, and anticoagulants [4]. Notably, diverticulitis was not identified as a potential cause in either review [3, 4].

Given the rarity of ASR, its risk factors and natural history require further study. However, splenomegaly has been identified in more than half of cases [3]. The mortality rate from ASR is ~12% to 20%; therefore, it should be considered a life-threatening emergency [3, 7, 8]. Splenomegaly, neoplastic disorders, age older than 40 years, and delayed diagnosis are potential risk factors for ASR-related mortality [3, 9]. The majority of patients with ASR undergo splenectomy within 24 h of diagnosis, with only 1% and 15% receiving organ-preserving surgery or non-operative treatment, respectively [3, 10]. In contrast, ~50% to 70% of cases of splenic rupture secondary to trauma can be managed non-operatively [6]. Operative or non-operative management of ASR should be determined based on the degree of splenic injury and hemodynamic stability of the patient [8].

The mechanism of ASR secondary to diverticulitis is likely similar to that of pancreatitis, where inflammation occurs in close proximity to the spleen [11]. Neutrophil infiltration and edema may lead to vascular damage, increased pressure within the spleen, hemorrhage, and capsular tear [11, 12]. In our case, the diverticular perforation occurred directly onto the splenic capsule.

To our knowledge, only two cases of ASR in the setting of diverticulitis have been previously reported [12, 13]. One case involved perforated jejunal diverticulitis and subacute pancreatitis [13]. The other case involved complicated sigmoid diverticulitis with a pelvic abscess [12]. Interestingly, in all three cases, the patients were discharged after attempted non-operative management of diverticulitis before representing with ASR.

Although diverticulitis is an extremely rare cause of ASR, clinicians should be aware of this possible association to enable its timely diagnosis and treatment. ASR should be considered in the setting of diverticulitis in proximity to the spleen and sudden, severe abdominal pain.
